# Radiological findings in patients undergoing revision endoscopic sinus surgery: a retrospective case series study

**DOI:** 10.1186/1472-6815-11-4

**Published:** 2011-05-07

**Authors:** Hisham S Khalil, Ahmed Z Eweiss, Nicholas Clifton

**Affiliations:** 1 Department of Otolaryngology, Derriford Hospital, Plymouth, U.K. and Faculty of Medicine, University of Alexandria, Egypt; 2 Department of Otolaryngology, Derriford Hospital, Plymouth, UK

**Keywords:** Functional endoscopic sinus surgery, rhinosinusitis, revision FESS, sinus C.T scan, uncinate process

## Abstract

**Background:**

Functional endoscopic sinus surgery (FESS) is now a well-established strategy for the treatment of chronic rhinosinusitis which has not responded to medical treatment. There is a wide variation in the practice of FESS by various surgeons within the UK and in other countries.

**Objectives:**

To identify anatomic factors that may predispose to persistent or recurrent disease in patients undergoing revision FESS.

**Methods:**

Retrospective review of axial and coronal CT scans of patients undergoing revision FESS between January 2005 and November 2008 in a tertiary referral centre in South West of England.

**Results:**

The CT scans of 63 patients undergoing revision FESS were reviewed. Among the patients studied, 15.9% had significant deviation of the nasal septum. Lateralised middle turbinates were present in 11.1% of the studied sides, and residual uncinate processes were identified in 57.1% of the studied sides. There were residual cells in the frontal recess in 96% of the studied sides. There were persistent other anterior and posterior ethmoidal cells in 92.1% and 96% of the studied sides respectively.

**Conclusions:**

Analysis of CT scans of patients undergoing revision FESS shows persistent structures and non-dissected cells that may be responsible for persistence or recurrence of rhinosinusitis symptoms. Trials comparing the outcome of conservative FESS techniques with more radical sinus dissections are required.

## Background

Functional endoscopic sinus surgery (FESS) has become a well established strategy for the treatment of rhinosinusitis not responding to medical treatment [1]. Published success rates for FESS vary from 76% to 98% [2]. However, there remains a group of patients in whom FESS does not provide symptomatic relief [3]. Some of these patients may require revision FESS. In a national audit of the sinonasal surgery in the UK, it was shown that 11.4% of patients had revision surgery within 3 years of the primary procedure [4].

Revision endoscopic sinus surgery represents a challenge to all who practise sinus surgery. Among the most important considerations in revision sinus surgery is the identification of the anatomy that is contributing to the patient's symptoms and the disease process [5].

A patient with persistent chronic sinusitis or recurrent infections after primary sinus surgery needs aggressive treatment with antibiotics and steroids. If, despite sufficient medical treatment, the patient's symptoms persist, a C.T scan is obtained to identify the source of infection. Once an anatomic aetiology of the primary surgical failure is identified, revision surgery is usually indicated [6].

Several anatomic findings have been identified in revision sinus surgery including a remnant of the uncinate process obstructing the maxillary ostium, residual ethmoidal partitions, lateralised middle turbinate and scarring of the frontal recess [5]. In the current study we have attempted to identify the anatomic factors that may be related to residual or recurrent sinus disease, as reflected on the C.T scans of patients admitted for revision FESS.

## Methods

**Figure 1:**
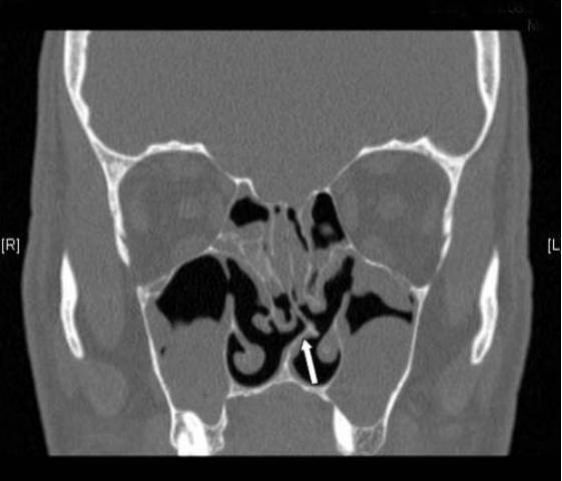
**Residual septal deviation**. A coronal C.T scan of a patient admitted for revision FESS showing a residual significant septal deviation (arrow).

**Figure 2:**
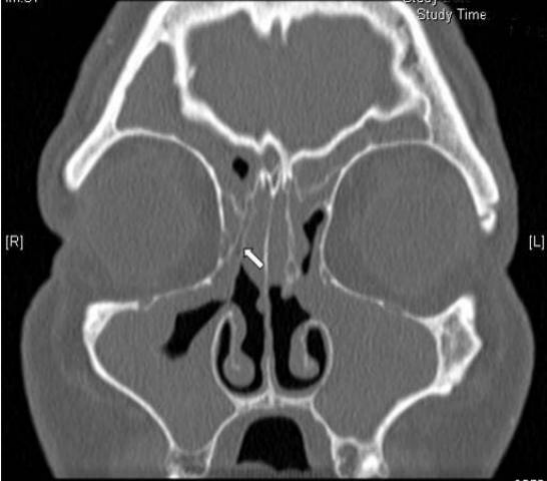
**Lateralised right middle turbinate**. A coronal C.T scan of a patient admitted for revision FESS showing a lateralised right middle turbinate (arrow).

The axial and coronal C.T scans of 63 patients admitted for revision FESS between January 2005 and November 2008 under care of the senior author (HSK) were retrospectively reviewed as a part of an audit of the outcomes of FESS. Some of the primary procedures had been performed in the authors' hospital, a tertiary referral centre in South West England, and some had been performed in other U.K hospitals and were referred to the senior author for revision surgery. All patients presented with symptoms and endoscopic findings of recurrent sinusitis that did not respond to medical treatment. All patients had had bilateral FESS and were all listed for bilateral revision FESS. The data collated included identification of significant septal deviation, middle turbinate lateralisation, residual uncinate process, residual Haller (infraorbital) cells, residual cells in the frontal recess, residual other anterior or posterior ethmoidal cells and condition of the sphenoid sinus ostium. The nasal septum was considered to be significantly deviated when the distance between the summit of the convex part of the septum and the lateral nasal wall was less than the distance between the summit of the convexity and the midline (figure 1). The middle turbinate was considered lateralised when it was close enough to the lamina papyracea to interfere with the sinus drainage pathways in the middle meatus (figure 2).

The mucosal disease of the paranasal sinuses and the ostiomeatal complex status were scored according to the Lund - Mackay staging system [7], where a sinus with no opacification is given a score of zero, a sinus with partial opacification is given a score of 1 and a sinus with full opacification is given a score of 2. A patent ostiomeatal complex is given a score of zero, while a blocked one is given a score of 2.

This study was registered as an audit in our hospital audit department, and thus no approval was required from the ethics committee.

## Results

The patients' ages ranged from 20 to 76 years, with a mean age of 50.3 years (+/- 12.1). There were 45 males and 18 females. As all patients had bilateral surgery, a total of 126 sides of paranasal sinuses were studied on the scans. The following results were identified:

The septums were significantly deviated in 10 patients (15.9%).

The middle turbinates were lateralised in 14 sides (11.1%).

Residual uncinate processes were identified in 72 sides (57.1%)

Residual Haller cells were identified in 29 sides (23%).

Residual frontal recess cells (Agger nasi and/or frontoethmoidal cells) were identified in 121 sides (96%).

Residual other anterior ethmoidal cells were identified in 116 sides (92.1%).

Residual posterior ethmoidal cells were identified in 121 sides (96%).

Blocked sphenoid sinus ostia were identified in 83 sides (65.9%).

The anterior ethmoidal and the frontal were the sinuses most frequently showing total opacification on the scans. Each of these 2 sinuses was totally opacified in 67 of the studied sides (53.2%).

The maxillary was the sinus least frequently showing total opacification, being totally opacified in 41 of the studied sides (32.5%).

The sphenoid was the most frequent sinus to show no opacification. This was detected in 36 of the studied sides (28.6%).

The maxillary was the least frequent sinus to show no opacification. This was found in only 3 of the studied sides (2.4%).

The ostiomeatal complex was patent in 21 of the studied sides (16.7%), and was blocked in 105 sides (83.3%).

**Figure 3:**
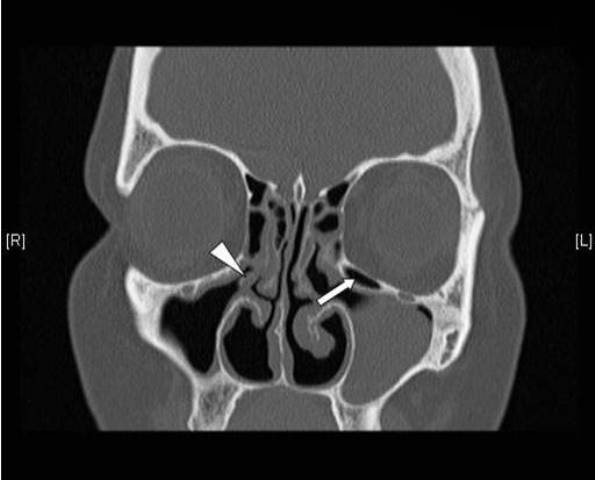
**Residual uncinate process and Haller cell**. A coronal C.T scan of a patient admitted for revision FESS showing a residual right uncinate process (arrow head) and a residual left Haller (infraorbital) cell (arrow).

**Figure 4:**
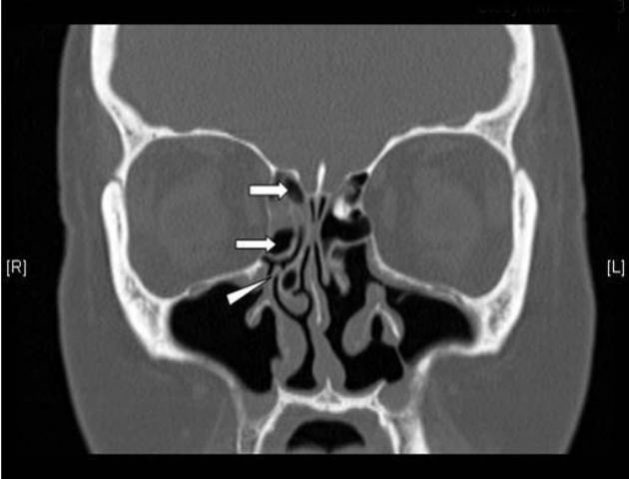
**Residual uncinate process and anterior ethmoidal cells**. A coronal C.T scan of a patient admitted for revision FESS showing a residual right uncinate process (arrow head) and residual anterior ethmoid cells (arrows).

**Figure 5:**
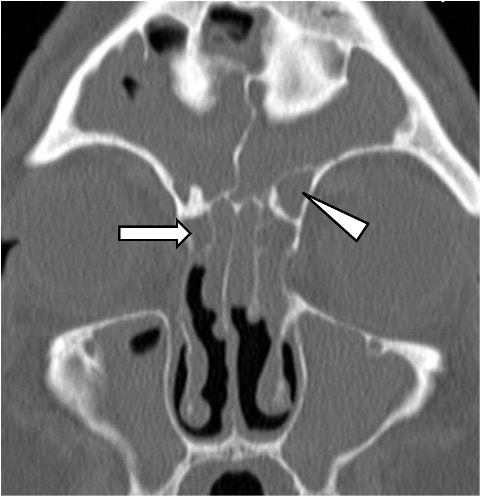
**Residual frontal recess cells**. A coronal C.T scan of a patient admitted for revision FESS showing a residual right agger nasi cell (arrow) pneumatising within right frontal recess and a residual left type III frontal cell (arrow head) pneumatising from the left frontal recess into the frontal sinus and partly obstructing the frontal sinus ostium.

**Figure 6:**
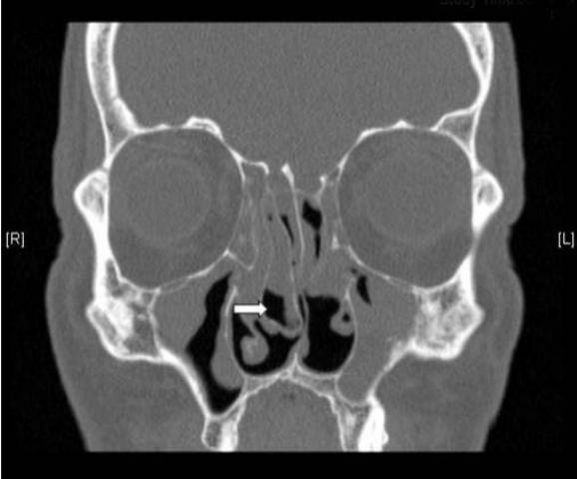
**Residual large right concha bullosa**. A coronal C.T scan of a patient admitted for revision FESS showing a residual large right concha bullosa (arrow).

Table 1 summarises the incidence of anatomical abnormalities identified on the CT scans of the paranasal sinuses. Figures 1, 2, 3, 4, 5 and 6 demonstrate the abnormalities identified on the CT scans.

**Table 1:** Anatomical abnormalities

Anatomical Abnormality	Incidence in 63 patients	Incidence in 126 studied sides
Septal Deviation	10 (15.9%)	

Lateralized Middle Turbinate	11 (17.5%)	14 (11.1%)

Residual Uncinate Process	38 (60.3%)	72 (57.1%)

Residual Haller Cells	16 (25.4%)	29 (23%)

Residual Frontal Recess Cells	61 (96.8%)	121 (96%)

Residual Anterior Ethmoidal Cells	58 (92.1%)	116 (92.1%)

Residual Posterior Ethmoidal Cells	61 (96.8%)	121 (96%)

Obstructed Sphenoid Sinus Ostium	43 (68.3%)	83 (65.9%)

Anatomical abnormalities in the CT scans of the paranasal sinuses of 63 patients admitted for bilateral revision FESS.

Table 2 summarises the mucosal status of the paranasal sinuses as assessed from the C.T scans.

**Table 2:** Mucosal status of the paranasal sinuses

Sinus involved	Number of sides with no opacification	Number of sides with partial opacification	Number of sides with total opacification
Anterior ethmoids	9 (7.1%)	50 (39.7%)	67 (53.2%)

Posterior ethmoids	9 (7.1%)	61 (48.4%)	56 (44.4%)

Maxillary	3 (2.4%)	82 (65.1%)	41 (32.5%)

Frontal	28 (22.2%)	31 (24.6%)	67 (53.2%)

Sphenoid	36 (28.6%)	41 (32.5%)	49 (38.9%)

Ostiomeatal complex	21 (16.7%)		105 (83.3%)

The mucosal status of the paranasal sinuses of 63 patients admitted for bilateral revision FESS as evident on their C.T scans.

## Discussion

Several reasons have been identified for failure of primary FESS. Kennedy [8] noted that patients with bilateral ethmoid disease and additional disease in 2 or more dependant sinuses on each side, as well as patients with diffuse polyps, had significantly worse outcome after FESS than patients with less severe sinus disease. Lazar et al [9] found that fibrosis and adhesion formation, particularly between the middle turbinate and the lateral nasal wall, was the most common intraoperative findings in revision FESS. This was found in 43% of their patients. Recurrence of polyps was the second commonest finding, occurring in 22% of patients. Other causes for failure of primary FESS include lateralisation of the middle turbinate, frontal recess obstruction, recirculation between the natural ostium of the maxillary sinus and the antrostomy, persistent uncinate process, persistent agger nasi cells, severe septal deviations and devitalised bone [6].

Very few articles attempted to identify the incidence of the various anatomic findings in patients undergoing revision FESS. Musy and Kountakis [2] found that the most common anatomic factor associated with primary sinus surgical failure was lateralisation of the middle turbinate, occurring in 78% of their patients. Their results also showed residual anterior ethmoidal cells in 64% of patients. Scarred frontal recesses were found in 50% of patients. Residual posterior ethmoidal cells were present in 41% of patients. Residual agger nasi cells were present in 49% of patients. Residual uncinate processes were present in 37% of patients. Finally, middle meatal antrostomy stenosis was present in 39% of patients. In another study by Ramadan [10], the most common anatomic finding during revision FESS was adhesions, often involving a lateralised middle turbinate. This occurred in 56% of patients. This investigator also detected residual ethmoidal cells in 31%, middle meatal antrostomy stenosis in 27% and frontal sinus ostium stenosis in 25% of patients undergoing revision FESS.

In comparison with Musy and Kountakis' findings [2], the current study showed a noticeably higher incidence of residual cells. Our data show that 96% of the studied sides (96.8% of patients) had residual posterior ethmoidal cells, 96% of the sides (95.2% of patients) had residual frontal recess cells and 92.1% of the sides (92.1% of patients) had residual other anterior ethmoidal cells. On the contrary, lateralisation of the middle turbinate was only detected in 11.1% of the sides (17.5% of patients), which was significantly less than Musy and Kountakis' figure of 78% [2]. These results may reflect the more conservative FESS techniques practised by the majority of the surgeons in the U.K, in comparison with the practice in the U.S. It is of course to be argued that removal of all cells is not required in the majority of FESS procedures, and that the procedure has to be tailored to the extent of the pathology. However, the majority of the patients in the current study had pansinusitis, as can be seen from the Lund-Mackay [7] scoring of the involved sinuses, where only 2.4% of the maxillary sinuses, 7.1% of the anterior and posterior ethmoids and 22.2% of the frontal sinuses were non opacified. It is therefore reasonable to assume that the majority of these patients needed more aggressive surgical dissections than what they had during the primary surgery.

The current study showed that 57.1% of the sides (60.3% of the patients) had a residual uncinate process. Chiu and Kennedy [5] advised that identifying an uncinate process remnant was the most critical step in revising a middle meatal antrostomy. They also commented that residual Haller (infraorbital) cells could be a source of persistent obstruction of the maxillary sinus. The latter cells were found in 23% of the sides (25.4% of the patients) in the current study. However, recently some studies have advocated preservation of the uncinate process due to its role in protecting the sinuses from allergens and contaminated inspired air [11,12].

The above discussion highlights the fact that there is a wide variation in the practice of endoscopic sinus surgery. Some surgeons prefer more conservative techniques. Recently, the principle of minimally invasive sinus technique (MIST) has been introduced [13,14]. It is claimed that this entails a standardised conservative endoscopic technique that can be applied to all patients requiring sinus surgery, regardless of the extent of their pathology. Other authors, however, have disapproved of the principles of MIST [15]. On the other extreme, some surgeons prefer radical endoscopic surgical techniques to treat advanced inflammatory sinus pathology. Such techniques may involve total sphenoethmoidectomies with extensive mucosal resection [16], and may even involve middle turbinate resection as well [17-19].

We have not attempted in our study to investigate the clinical outcome after revision surgery as the aim of the study was to identify the residual anatomic factors that may result in recurrent rhinosinusitis after primary surgery, and to reflect on the practice of FESS in the U.K. We hope, however, that this work will stimulate further studies to compare conservative versus more aggressive sinus surgery techniques, and to answer the question of how extensive sinus surgery should be.

## Conclusions

Analysis of C.T scans of patients undergoing revision FESS demonstrates persistent anatomic structures and non dissected cells that may be responsible for persistence or recurrence of rhinosinusitis. Trials comparing the outcome of conservative FESS techniques with more radical sinus dissections are required.

## Competing interests

The authors declare that they have no competing interests.

## Authors' contributions

The first author (HSK) initiated the idea of the study. All patients were treated under his care. Both the first and the second authors reviewed the patients' scans and collated the data. The second author (AE) reviewed the literature and prepared the manuscript, which was revised by the first author. The third author (NC) reviewed the scans and helped making the revisions recommended by the reviewers. All authors have reviewed and approved the final manuscript.

## Pre-publication history

The pre-publication history for this paper can be accessed here:

http://www.biomedcentral.com/1472-6815/11/4/prepub
